# Climbing Accidents—Prospective Data Analysis from the International Alpine Trauma Registry and Systematic Review of the Literature

**DOI:** 10.3390/ijerph17010203

**Published:** 2019-12-27

**Authors:** Simon Rauch, Bernd Wallner, Mathias Ströhle, Tomas Dal Cappello, Monika Brodmann Maeder

**Affiliations:** 1 Institute of Mountain Emergency Medicine, Eurac research, Viale Druso 1, 39100 Bolzano, Italy; bernd.wallner@tirol-kliniken.at (B.W.); Monika.Brodmann@eurac.edu (M.B.M.); 2 Department of Anesthesia and Intensive Care Medicine, “F. Tappeiner” Hospital, Via Rossini 12, 39012 Merano, Italy; 3 Department of Anesthesiology, Medical University of Innsbruck, Anichstraße 35, 6020 Innsbruck, Austria; 4 Department of General and Surgical Critical Care Medicine, Medical University of Innsbruck, Anichstraße 356020 Innsbruck, Austria; mathias.stroehle@tirol-kliniken.at; 5 Department of Emergency Medicine, Bern University Hospital and Medical University of Bern, 3010 Bern, Switzerland

**Keywords:** climbing, trauma, accident, International Alpine Trauma Registry, literature review

## Abstract

Climbing has become an increasingly popular sport, and the number of accidents is increasing in parallel. We aim at describing the characteristics of climbing accidents leading to severe (multisystem) trauma using data from the International Alpine Trauma Registry (IATR) and at reporting the results of a systematic review of the literature on the epidemiology, injury pattern, severity and prevention of climbing accidents. We found that climbing accidents are a rare event, since approximately 10% of all mountain accidents are climbing related. Climbing accidents mainly affect young men and mostly lead to minor injuries. Fall is the most common mechanism of injury. Extremities are the most frequently injured body part. However, in multisystem climbing-related trauma, the predominant portion of injuries are to head/neck, chest and abdomen. The fatality rate of climbing accidents reported in the literature varies widely. Data on climbing accidents in general are very heterogeneous as they include different subspecialties of this sport and report accidents from different regions. A number of risk factors are accounted for in the literature. Appropriate training, preparation and adherence to safety standards are key in reducing the incidence and severity of climbing accidents.

## 1. Introduction

Rock climbing originated in the 1960s from mountaineering and was, at that time, practiced by a small group of dedicated mountaineers only. Over the following decades, its popularity spread globally and climbing diversified to include new categories such as ice climbing, bouldering (ropeless climbing to low heights), speed climbing (competition climbing where two climbers climb simultaneously on identical routes against each other) or free solo climbing (climbing alone and without rope). In 1991, the first World Championships took place, with only a few countries participating. However, in 2005, some 500 athletes from 55 countries competed and in 2020, climbing will make its Olympic debut in Tokyo [1,2,3,4,5]. In parallel to the increasing popularity of this sport, the number of climbing accidents and emergency department consultations due to climbing-related injuries has reportedly increased [2,6]. In a study from Colorado, rock climbing rescues accounted for approximately 20% of mountain and wilderness rescue victims [7]. Climbing accidents usually affect a young and healthy population.

When assessing climbing-related injuries, a distinction between overuse (overstrain) injuries and acute injuries or accidents should be made. Overuse injuries are generally less severe and can be avoided with informed training in many cases [5]. Acute injuries, on the other hand, can range from minor to life threatening, involve a single body part or multiple body regions and usually cannot be avoided by training. A considerable amount of studies has been published on climbing-related overuse injuries or acute injuries involving a single body part, e.g., the fingers or shoulder. However, scant data exist on accidents involving multiple body regions with regard to the cause of injury, injury mechanism and accident characteristics, injury pattern and preventive measures. To the best of our knowledge, no data have been published on severe (multisystem) climbing-related trauma. Yet, information on the injury mechanism and injury pattern is fundamental to improve preventative injury measures and provide safety precautions. This study, therefore, aimed to analyze the characteristics of climbing accidents leading to multisystem trauma recorded in the International Alpine Trauma Registry (IATR) and report the results of a systematic review of the literature on the epidemiology, injury pattern, severity and prevention of climbing accidents leading to acute injuries. 

## 2. Materials and Methods 

### 2.1. International Alpine Trauma Registry (IATR)

The IATR is a transnational platform for the prospective collection and storage of data related to multisystem trauma patients encountered in mountainous or remote areas that are not readily accessible by the regular emergency medical services. Multisystem trauma is defined as trauma with an injury severity score (ISS) ≥16. Data collection in the IATR is based on the Utstein style [8], which requires comprehensive data collection of multiple parameters. These parameters include accident and mission characteristics plus timing. Medical data comprise vital signs on the scene (i.e., systolic blood pressure, respiratory rate, Glasgow coma scale (GCS) and body temperature). Out-of-hospital advance trauma life support (ATLS) interventions recorded in the IATR include endotracheal intubation, intravenous or intraosseous cannulation as well as fluid and drug administration. Parameters collected on hospital admission include vital signs and laboratory values (i.e., hemoglobin, international normalized ratio (INR), base excess) as well as a classification of abbreviated injury scale (AIS) based on in-hospital diagnosis and calculation of the ISS. The data collection also includes out-of-hospital and in-hospital mortality. The registry is hosted in Bolzano, Italy, and, by the time of publication, the following regions/centers have been participating: Italy (Hospitals of Bolzano, Merano, Bressanone, Brunico, Silandro, Vipiteno, San Candido, “Umberto Parini” Hospital Aosta), Austria (University Hospital Innsbruck) and Switzerland (Hospital of Graubünden, Chur). This study included data from December 2010 to June 2013 and from August 2014 to June 2019 (data collection was temporarily interrupted from June 2013 to August 2014 due to technical reasons).

### 2.2. Literature Search

An electronic PubMed query was performed in September 2019 using the key words: “Rock Climbing AND Accident” (43 hits), “Rock Climbing AND Crash” (1 hit), “Rock Climbing AND Injury” (141 hits). Preferred Reporting Items for Systematic Reviews and Meta-Analyses (PRISMA) guidelines were used for literature search strategy, selection of publications, data extraction, and reporting of results for the review. Thirteen additional articles were found in references and added to the analysis. We excluded duplicates and papers written in a language other than English. We did not limit the search by year of publication. The papers were crosschecked by authors (SR, MBM, BW), and full text was assessed for eligibility. Included were original articles on rock climbing accidents and associated injuries, analyzing acute injuries involving the entire body. Exclusion criteria were review articles, studies on specific injuries of a single body region, chronic pathologies resulting from rock climbing, accidents resulting from indoor climbing and case reports. The rock-climbing ability grade was converted into the Union Internationale des Associations d’Alpinisme (UIAA) scale and units were converted into metric scale [9]. Data from eligible studies were extricated independently by authors (SR, MBM, BW) and inserted into an Excel sheet. 

## 3. Results

### 3.1. Climbing Accidents Recorded in the IATR

The IATR recorded a total of 306 mountain accidents leading to a multisystem trauma—of which, 37 (12.1%) were climbing accidents. Mean patient age was 44.1 ± 14.9 years; 27 (72.9%) were male and the majority (81.1%) were previously healthy (American Society of Anesthesiologists Classification, ASA, class I). 

Most accidents (57.8%) occurred during summer months (July, August, and September). Fourteen (38.9%) accidents occurred in the morning hours (from 06:00 to noon), 19 (52.8%) in the afternoon (from noon to 18:00) and three (8.3%) in the evening (from 18:00 to 22:00); in one case, the time of the accident was not recorded. 

Fall onto the ground was the most common mechanism leading to trauma (51.4%), followed by fall in a snowfield (10.8%), fall in a crevasse (8.1%) and hit by a stone (5.4%). In nine cases (24.3%), the cause was unknown. The mean height of fall was 31.4 ± 56 m (median 17.5 m). In 12 patients (32.4%) the accidents occurred while they were being belayed. Three (8.1%) did not wear a harness.

In almost half of the cases (48.6%) the terrain was exposed and in 40.5% of cases belaying the rescue team was necessary. In 94.6% of the accidents, the helicopter was involved in the rescue mission (air rescue only in 59.5% of cases, mixed air-terrestrial rescue in 35.1%). Only in 5.4% the rescue mission was performed terrestrially.

Clinical characteristics on scene are reported in Table 1. On site, most patients (86.5%) received analgesic therapy, either ketamine (43.2%) or opioids (62.1%), 21.6% received both. Twenty-three (62.2%) patients received volume replacement on the scene—of which, eight (34.7%) patients received crystalloids only, one (4.3%) hypertonic solution only and 14 (60.9%) patients a combination of crystalloid and colloidal solutions (information missing in four patients). The mean volume of infusion was 600 ± 300 mL for crystalloids and 500 ± 0 mL for colloids. 

Clinical characteristics and selected laboratory values on hospital arrival are reported in Table 1. Upon arrival, 15 invasive, emergency procedures were performed: chest tube insertion and intracranial surgery in five patients each, surgical/radiological intervention for pelvic bleeding control in four and emergency surgery for bleeding control other than pelvic bleeding in one patient. 

The mean ISS was 29.6 (±11.1), the median ISS 28. Injury pattern and severity assessed in hospital according to the abbreviated injury severity (AIS) of the different body regions is shown in Table 2.

Thirty-one patients (83.8%) survived until hospital discharge, four patients (10.8%) died in hospital (for two patients, information on outcome is missing). Among the survivors, 16 (51.6%) survived with good neurologic outcome, 12 (38.7%) had moderate disability and three (9.7%) suffered severe disability. 

### 3.2. Literature Review 

#### 3.2.1. Injury Severity and Injury Pattern

In our literature review, we identified two articles reporting injury severity and pattern of injury in acutely injured climbers. 

Forrester et al. performed a retrospective analysis of emergency department (ED) visits in the US from 2010 to 2014 [10]. They reported a weighted estimate of 15,116 adult ED visits associated with climbing-related injury. The authors found that 60% of climbing accidents led to injuries at multiple body regions. Among patients with isolated injuries, the extremities were by far the most commonly injured body region (32%), followed by head/neck (5%), abdomen (2%), chest (1%). Two percent of their patients had an ISS score >15; <1% of them died following the climbing accident. 

In a European study, Hohlrieder et al. identified a total of 113 climbers injured after a fall equal or exceeding 5 m from 2000 to 2004 [11]. Eighty-three (73.5%) climbers were uninjured or had minor injuries, 17 (15.0%) sustained moderate injuries, and 13 (11.5%) had severe or critical multisystem trauma. Fractures and dislocations of the lower extremities and the pelvic region were the most common injuries (22.1%), followed by trauma of the upper extremity and shoulder (15.0%), head (13.3%), chest (8.0%) and spine (7.1%). The most severe injuries occurred in the head and neck region. The mean ISS was 6.2 (range 1–41). 

#### 3.2.2. Epidemiology

The number of rock climbers presenting to US Emergency Departments for rock climbing-related injuries ranges from 3023 (±149) [10] to 3816 (±854) [12] per year. Schussmann et al. found an incidence of 2.5 accidents per 1000 mountaineers per year or 5.6 injuries per 10,000 hours of mountaineering [13].

According to Lack et al. most rock climbing incidents occurred on weekends (median time 3:30 pm) during spring, summer, and autumn [6].

#### 3.2.3. Demographics

In all studies analyzed, male climbers accounted for the majority of injured patients (range 66–88%) [7,12,14,15]. Bowie et al. further found that more experienced climbers (mean 5.9 years of climbing experience) were injured more often. Schoffl et al. stated that male climbers were significantly older, had more climbing years, and were climbing at a higher climbing level [5].

In rock climbing accidents, the younger population is predominantly represented. Nelson et al. stated that patients aged 20–39 years accounted for more than half of all injured [2]. According to Buzzacott et al., the age group from 20–39 years account for 60% of injured rock climbers [12]. Forrester et al. described a mean age of 32.8 (±14.7) years [10].

#### 3.2.4. Causes of the Accidents

A number of papers analyzed the causes of rock-climbing accidents. Falls accounted for the majority of cases in traditional climbing (75% reported by Addis et al.) [9], while in sport climbing, strenuous moves tended to cause most injuries [16,17].

#### 3.2.5. Fatality of Accidents 

The fatality rate from rock climbing accidents varies quite significantly: Forrester et al. found a mortality rate of <1% [10], Lack et al. reported 5.5% [7], Bowie et al. 6% [14] and Schussmann et al. 20% [13], whereas Ferris et al. reported a fatality rate of 41% [18]. Falls on snow or ice or during ice climbing were more likely to be fatal [9,14,17,19,20].

#### 3.2.6. Climbing-Related Injuries Cost

Forrester et al. were the only to describe the cost of climbing-related injuries to the US health care system, which are approximately 102 (95% CI: 75–130) million USD for the 5 year period, averaging 20 ± 9.5 million USD per year. More than one-tenth of patients required hospital admission as inpatients after a climbing accident [10,21].

#### 3.2.7. Risk, Risk Factors and Prevention

The first literature review on risk assessment, risk factors and prevention specifically revealed only four articles. Another sixteen articles could be found by cross referencing. 

Several publications aim to stratify the injury and fatality risk of climbing and compare them with other sports. Schöffl et al. performed a review on injury and fatality risks in different types of climbing and compared them with various sports [5]. They found traditional climbing more than 20 years ago to have an injury risk of 37.5 injuries per 1000 hours of sport performance, which was higher than the injury risk of American football (15.7) or motorbiking (22.4). Another publication by Schussman et al. from 1990 reported a much lower risk of mountaineering (0.56) [20]. Ice climbing had an injury risk of 4.07 [22], competition climbing 3.1 [23], and indoor climbing had the lowest injury risk of 0.027 [24]. Backe et al. in 2009 reported an overall injury risk for rock climbing of 4.2, but did not differ between the various climbing subspecialties [25]. Most of them (93%) were overuse injuries affecting fingers and wrists of the climbers. Neuhof et al. studied acute sport climbing injuries and found an injury risk of 0.2 for sport climbing [21]. Other authors such as Josephsen or Paige, define risk as the incidence or prevalence of career injuries [17,26]: Josephsen et al. performed a prospective investigation on 53 boulderers and found an overwhelming amount of injuries while bouldering [26]. Most of the injuries were minor and affected fingers or shoulders. The authors compared their numbers with career injury incidences of around 50% for traditional and sport climbers [12]. Main reasons for acute injuries were falls and strenuous moves [13]. According to Addiss et al. the length of the fall is an important risk factor. They reported that falls that were survived had a mean height of 9 m. In comparison, falls that were not survived were from a mean height of 91 m [9]. Falls on snow or ice were longer than falls on rock [9,14,17,19]. 

The majority of injuries (Addiss et al., 69%) occurred while ascending as a lead climber [9]. Technical roped climbers accounted for 58% of climbing victims, unroped climbers accounted for 34%, belay incidents accounted for 12% of climbing victims, and rock fall incidents accounted for 4.5% of victims [7].

Individual risk factors are discussed contradictorily. Authors list male sex, younger age, more than ten years of experience, overweight, the use of illicit substances and alcohol as risk factors for injuries during climbing [5,9,15]. Injuries happen during lead climbing or are caused by belay mistakes [16]. Preventive measures are rarely reported. They contain training, taping of fingers and wrist or the use of a helmet [11]. As falls are frequent during bouldering, the use of safety mattings and the presence of a spotter are strongly recommended [11].

## 4. Discussion

The present study found that climbing accidents are generally a rare but potentially fatal occurrence affecting predominantly young men. Fall is the most common injury mechanism. Extremities are the most commonly injured body part. In severe, multisystem climbing-related trauma, injuries to the head/neck, chest and abdomen prevail. A number of risk factors have been identified. However, options for prevention have rarely been analyzed in the literature.

The incidence of climbing accidents varies widely amongst published studies, depending on the country/region and on the subspecialty of climbing. Publications include reports on sport climbing, rock climbing and mountaineering, bouldering, ice climbing, and speed, solo or free climbing. Free solo climbing is performed without any safety device and is the most dangerous form of climbing. Bouldering, on the other side, is usually performed a few meters above the ground, uses special matting and so-called “spotters” (i.e., a partner on the ground who reduces the impact of an unprotected fall) and the injuries sustained are usually minor and mostly affect fingers and upper extremities [26]. Injury frequency and severity differs largely in these two examples of subdisciplines, but they are all summarized as sport climbing. In addition, climbers often do not seek a doctor’s advice and perform self-therapy. Gerdes et al. found that only one-third of the climbers sought medical treatment [16], which makes it even more difficult to gather exact numbers on the incidence, especially of accidents leading to minor injuries. The German Alpine Club reports 940 mountain accidents in 2017, and 96 (10.2%) of them were related to climbing [27]. The Swiss Alpine Club accounted for 144 emergencies during climbing in the mountains in 2018, compared to 1445 emergencies during alpine hiking [28]. However, these numbers must be seen in the context of the number of persons who are climbing compared to the many more persons who are hiking. In the IATR, which only includes patients who sustained a multisystem trauma (with an ISS > 16) in the mountains, approximately one in ten accidents occurred during climbing. The risk of injury differs between the subspecialties of climbing: Indoor climbing seems to have the lowest risk of injury (0.02 per 1000 climbing hours) [29] whereas bouldering is associated with the highest injury risk of more than 80% career prevalence [26].

The majority of acute climbing accidents are due to falls, as reported by the data from the IATR and confirmed in several previous publications. Falls on snow and ice were reportedly longer than falls on rock and in most studies, the height of fall correlated with the injury severity [9,14,17,19]. Strenuous movements are generally the second most common cause of injury reported in the literature. However, this mechanism of injury usually results in a localized lesion to muscles or tendons [16,17]. Most climbing accidents lead to minor injuries. Hohlrieder et al. found that more than two-thirds of the climbers were uninjured or suffered only mild injuries and the mean ISS in that study was 6.2 [11]. Two studies form the US found that only approximately one in ten patients seen in the emergency department because of a climbing-related injury requires hospital admission and treatment as an inpatient [10,21]. An ISS of > 15 was documented in 11.5% of cases by Hohlrieder et al. [11] and in merely 2% in the study by Forrester et al [10]. The most frequently affected body region in mildly injured patients is the upper [5,16] or lower extremity [14]. Injury pattern changes in more severely traumatized patients. In the IATR, the mean ISS was 29.6 and the chest, the head and neck and the abdomen are more frequently affected. Moreover, severely injured climbers are often hypothermic. The mean body temperature of the patients from the IATR was approximately 35 °C. Hypothermia is an independent risk factor for mortality in trauma patients [30] and the prevention of hypothermia is key and can also be achieved by companions while awaiting professional rescue. Rescuing multisystem trauma patients proves to be a challenge. The IATR data analysis showed that the terrain is often difficult and exposed. In almost all cases, helicopter rescue was necessary and frequently (40.5%) belaying of the rescue team was mandatory. In multisystem trauma patients, advanced trauma life support such as intravenous cannulation with administration of fluids and analgesic therapy was necessary in most of the cases. 

According to literature found on the number of climbing accidents and fatality rate, mountain climbing cannot be defined as high-risk sport, except for free solo climbing, where a fall is often fatal. The annual report on mountain emergencies by the Swiss Alpine Club confirm this statement [28]: the secular trend counts five fatalities per year during climbing in Switzerland, compared to a mean of 122 fatal injuries during mountaineering. Similar data are reported from Austria. The registry of the Austrian Alpine Safety Board is one of the largest registries for mountain accidents and from 2008 to 2018, a mean of 284 fatal mountain accidents was documented. Of these, 17 (6.0%) per year are due to rock (16) or ice (1) climbing [31]. However, the huge heterogeneity of studies constitutes a challenge to identify robust data on injury and fatality risk in climbing. Most of the articles are retrospective or describe the prevalence of climbers who received medical treatment [2,32]. Moreover, injury and fatality risk of different (high risk) sports are differently reported: the injury and fatality risks of BASE jumping, for example, are expressed as the number of incidences per 1000 jumps [33].

Several risk factors for climbing accidents are documented in the literature, such as unroped climbing [7], ascending as a lead climber [9], overweight and climbing after alcohol or illicit drug abuse [16]. A high level of experience is reported as a risk factor for climbing accidents [14]. Apart from climbing-specific risks, outdoor climbing comprises additional risks such as rockfall, wet, snowy or icy surface, low temperature, high altitude, damaged pitons or anchors. These environmental factors are eliminated in indoor climbing, resulting in fewer accidents. Equipment failure was not mentioned in previous studies [20]. Yet, human factors, e.g., belay mistakes play a major role in the causation of an injury. In the study by Lack et al, belay incidents accounted for 12% of climbing injuries [7]. Therefore, a systematic partner check before starting a climb could improve safety and must be an essential part of climbing training courses, as advised by the safety standards of the International Climbing and Mountaineering Foundation UIAA [34]. They provide safety labels for materials that meet their safety standards and provide recommendations for belaying techniques or the use of bolts and carabiners. Preparation for climbing should cover individual training, selection of routes that correspond to the climbing level of all climbers in a group, check of weather and local conditions, abdication of illicit drugs and alcohol during the activity and performing a partner check directly before the start of climbing. 

## 5. Limitations

The main limitation of the systematic literature search is the heterogeneity of the studies. The studies vary in the subtype of climbing analyzed, the study design (prospective or retrospective, pre-hospital or in-hospital data collection, etc.) and the injury of interest (acute or chronic, single body region or global injury pattern, etc.). Moreover, risk is either defined as the number of injuries per 1000 h of climbing activity, as incidence in a special cohort of climbers or as career prevalence. It is thus very difficult to provide an overview and exact numbers on epidemiology, injury severity and pattern or risk for the different climbing activities. For future research on the topic, a more differentiated approach based to the different subspecialties should be adopted, and research on risk stratification should follow more uniform guidelines, as already required many years ago [16].

Albeit the prospective design, data in the IATR are often incomplete. This is a common problem of registries, particularly in emergency medicine. However, due to the overall low incidence of mountain trauma, international registries are the only way to increase our knowledge and the quality of evidence in this field. 

## 6. Conclusions

Climbing accidents account for approximately 10% of all mountain sport-related accidents and men in their thirties and forties are mainly affected. Climbing accidents lead to minor injuries in the majority of cases, and the extremities are most commonly affected. In more severe trauma, injuries of the head and neck, chest and abdomen prevail. However, numbers on incidence, injury severity and fatality rate vary widely due to the large heterogeneity in literature review. Appropriate training and preparation and adherence to the safety standards are key in reducing the incidence and severity of climbing accidents. To further improve preventative measures, more standardized and sport-specific research is needed to report and fully evaluate the injury risk, injury mechanism, the severity of injuries and fatality risk in climbing sports.

## Figures and Tables

**Table 1:** Clinical characteristics on the scene and on hospital arrival plus selected laboratory values on hospital arrival.

**Clinical Characteristics on Scene**	**Mean ± SD**	**Information Missing**	
Glasgow coma scale	12.8 ± 3.0	4	
Systolic blood pressure (mmHg)	120.3 ± 26.7	16	Of the 16 patients with missing exact systolic blood pressure: Good radial pulse—7 Week radial pulse—3 Carotid/femoral pulse only—2 Information missing—2
Body temperature (°C)	34.8 ± 1.0	27	
**Clinical characteristics on hospital arrival**	**Mean ± SD**	**Information missing**	
Glasgow coma scale	11.1 ± 4.8	1	
Systolic blood pressure (mmHg)	123.0 ± 23.8	1	
Body temperature (°C)	35.3 ± 1.4	8	
Hemoglobin (g/dL)	12.5 ± 2.1	1	
International normalized ratio (INR)	1.3 ± 0.4	1	
Base excess (BE)	−3.2 ± 5.9	4	

**Table 2:** Injury pattern and severity. Abbreviated injury scale (AIS).

Body Region	Number (%) of Injured Patients	Mean AIS Score (±SD)
Head and neck	25 (67.6)	3.1 (±1.1)
Face	10 (27.0)	2.8 (±0.6)
Chest	27 (73.0)	3.4 (±0.7)
Abdomen	18 (48.6)	3.4 (±1.1)
Extremities	14 (37.8)	3.4 (±0.9)
External	5 (13.5)	1.8 (±0.4)
